# Developmental Origins of Type 2 Diabetes in Aboriginal Youth in Canada: It Is More Than Diet and Exercise

**DOI:** 10.1155/2012/127452

**Published:** 2012-01-12

**Authors:** Kyle Millar, Heather J. Dean

**Affiliations:** ^1^Faculty of Medicine, University of Manitoba, Winnipeg, MB, Canada R3E 0Z2; ^2^Section of Endocrinology and Metabolism, Department of Pediatrics, University of Manitoba, Winnipeg, MB, Canada R3E 0Z2

## Abstract

Type 2 diabetes mellitus (T2DM) is classically viewed as a disease of adults caused by poor nutrition, physical inactivity, and obesity. However, with increasing awareness of the heterogeneity of T2DM, new risk factors are being identified that add complexity. Some of these new risk factors have been identified in Canadian people with Aboriginal Oji-Cree heritage, a group that demonstrates one of the highest rates of T2DM in the world. This high prevalence may be due to the rapid change, over the past 50 years, away from their traditional way of life on the land. Another environmental change is the increased rate of pregnancies complicated by obesity, gestational diabetes, or T2DM, resulting in more children being exposed to an abnormal intrauterine environment. Furthermore, the Oji-Cree of central Canada possesses the unique HNF-1**α** G319S polymorphism associated with reduced insulin secretion. We propose that intrauterine exposure to maternal obesity and T2DM, associated with the HNF-1**α** G319S polymorphism, results in fetal programming that accelerates the progression of early-onset T2DM. This paper describes the evolution of T2DM in children with a focus on the Oji-Cree people over the past 25 years and the unique prenatal and postnatal gene-environment interaction causing early-onset T2DM.

## 1. Introduction

The Diabetes Education Resource for Children and Youth (DER-CA), a regional pediatric diabetes ambulatory program at the Children's Hospital in Winnipeg, Manitoba, Canada, has been supporting youth with diabetes and their families since 1985. Although originally established to treat children with type 1 diabetes mellitus, the first unexpected presentation of type 2 diabetes mellitus (T2DM) in adolescents occurred in 1985. These youth were all of Aboriginal heritage and resided in Manitoba's remote northern communities. They matched the classic obese phenotype seen in adults with T2DM, with risk factors such as unhealthy diet and lack of physical activity [1]. However, as the number of youth with T2DM steadily increased over time, it was evident that some children were lean with minimal signs of insulin resistance such as acanthosis nigricans, but biochemically they had higher glucose levels, higher haemoglobin A1Cs, and lower serum insulin levels [1, 2]. The purpose of this paper is to review the evolution of T2DM in children in Manitoba in order to highlight the lessons learned and the impact of the intrauterine environmental on the risk of T2DM in children.

In a unique birth cohort started in 2003, roughly 50% of the children born to mothers who were diagnosed with T2DM in adolescence, and followed at the DER-CA, have developed T2DM before the age of 18 years old [3]. This unique observation and others related to the evolution of T2DM in youth in Manitoba over the past 25 years provide strong evidence to support the developmental origins of health and disease (DOHaD) theory [4]. The DOHaD theory suggests that organisms can adapt and develop in multiple ways in response to particular intrauterine or early-life experiences and that the origins of chronic disease may occur at the earliest times of life. The next generation of children with T2DM may have susceptibility to T2DM programmed into their development due to their early, intrauterine exposure to maternal obesity and T2DM. Today the Aboriginal youth of Manitoba have one of the highest incidence rates of T2DM in the world [5]. As the number of adolescent females with T2DM increases, there will be more pregnancies complicated by T2DM, and hence this vicious cycle will continue to accelerate the rates of T2DM in youth beyond what is predicted by obesity rates alone.

Contributing to the risk of T2DM, the Oji-Cree-speaking Aboriginal people of Northern Manitoba possess a private polymorphism in the hepatic nuclear transcription factor gene at codon 319 (HNF-1*α* G319S). The HNF-1*α* G319S polymorphism is associated *in vitro *with low insulin secretion, which allows expression of T2DM in the children who require physiological hyperinsulinemia to overcome differing degrees of insulin resistance [2, 6, 7]. This mechanism may explain why Oji-Cree children are being diagnosed with T2DM even without obesity or clinical signs of insulin resistance.

The expression of T2DM in these children may be further accelerated by other unknown insulin secretory defects or by a decrease in beta-cell mass perhaps caused by unique intrauterine or extrauterine epigenetic changes. Careful surveillance of the offspring of parents with adolescent-onset T2DM is required to describe the natural history of T2DM, to provide clinical evidence to support these hypotheses, and to uncover new mechanism and risk factors, as yet unidentified.

## 2. Type 2 Diabetes

Type 2 diabetes mellitus (T2DM) is a metabolic disorder characterized by insulin resistance, relative insulin deficiency, and subsequent hyperglycemia. The common comorbidities and complications of T2DM include hypertension, dyslipidemia, and microvascular disease resulting in end-organ damage to the kidneys, eyes, peripheral nerves, and heart. In Canada, diabetes is the most common cause of blindness, end-stage renal disease, and nontraumatic limb amputation. In addition, cardiovascular disease is 2–4 times more common in individuals with T2DM than in those without and is the main cause of death in individuals with T2DM in Canada [8]. Ten percent of all admissions to acute care hospitals in Canada in 2006-2007 were due to diabetic complications [9]. Compared with adults, youth with T2DM appear to have a more aggressive disease course with a greater probability of early-onset, T2DM-related complications [10].

## 3. The Experience of T2DM in First Nations Youth in Canada

Childhood T2DM is an emerging disease, with the prevalence of youth-onset T2DM paralleling the increasing trend seen in adults. T2DM with onset in childhood disproportionately affects indigenous populations and has now become a major health concern in these populations throughout the world [10]. The Canadian Aboriginal population consists of 3 groups: the Inuit people who were the original inhabitants of Canada's North, the Métis people who are of mixed European and First Nations heritage, and the First Nations people. The First Nations people compose 64% of the 1.2 million Aboriginal people in Canada, and nearly half (48%) are under the age of 25 [11]. In 2004–2006, the incidence of T2DM in Aboriginal youth (age < 18 years) was 23.2 cases per 100 000 in a total population of 215 831 Aboriginal youth [5]. However, there is a significant regional variation in incidence and prevalence of T2DM in Aboriginal youth across Canada [12].

## 4. The Experience of T2DM in Manitoban Youth

First Nations youth have the highest incidence of T2DM in the Canadian pediatric population, and the majority of these children reside in Manitoba, a province in central Canada [5]. The incidence of T2DM in children in Manitoba was 12.45 cases per 100 000 children in 2004–2006, compared to an overall rate of 1.56/100 000 children across Canada [5]. In the twenty-seven-year period between 1984 and 2011, the number of youth diagnosed with T2DM in Manitoba increased from 3 to 67 per year [1, 13]. In other regions of Canada with high proportions of Aboriginal people, the incidence of T2DM in children was lower, specifically 0.4/100 000 children in Saskatchewan and 0/100 000 children in the Northern territories [5, 14]. Thus, we hypothesize that unique risk factors for early-onset T2DM exist in Aboriginal children in Manitoba.

## 5. Risk Factors for T2DM in Manitoban Youth

### 5.1. Genetics

The Oji-Cree speaking people of Northern Manitoba and Northwestern Ontario experience one of the highest rates of T2DM in the world. In 1997 in Sandy Lake, an Oji-Cree community in Northwestern Ontario culturally and genealogically related to the Oji-Cree speaking people of Northern Manitoba, the crude prevalence of T2DM in all age groups was 17.2% [6]. In youth, aged 4–19 years, from a nearby Oji-Cree community, the prevalence of T2DM was 1.1% and in the adolescent females in this community the prevalence was 4% [12]. In part, the increased susceptibility of the Oji-Cree speaking people to T2DM is ascribed to the private HNF-1*α* G319S polymorphism [2, 7]. Knockout mice lacking any functional copy of HNF-1*α* have severe dysfunction in insulin production, the proposed diabetogenic mechanism of the S allele of HNF-1*α* [15]. In humans, the polymorphism results in two abnormal splicing products of the HNF-1*α* mRNA product, both of which result in premature termination codons, and an imbalance of the normal ratio of splicing products seen in wild-type HNF-1*α*. This may reduce the total concentration of HNF-1*α* mRNA transcripts and has been shown to decrease the *in vitro *ability of the HNF-1*α* protein product to activate transcription by roughly 50% with no effect on DNA-binding ability and protein stability [16, 17]. Upon clinical presentation, children and youth with T2DM and one or 2 copies of the G319S polymorphism are leaner, have lower insulin levels, greater insulin sensitivity, less acanthosis nigricans, and higher mean haemoglobin A1C than their peers with T2DM but without the G319S polymorphism [2]. This phenotype is consistent with decreased insulin secretion. Among individuals with T2DM in Sandy Lake, the possession of each additional copy of HNF-1*α* G319S accelerated the timing of disease onset by roughly 7 years [16]. In a 2002 cross-sectional study of all of the children with T2DM followed at the DER-CA, 41% had at least one copy of the G319S allele (GS or SS), and 78% of the children with T2DM from one remote regional Tribal Council (a collection of Aboriginal communities under a centralized government) in Northeastern Manitoba had at least one copy of G319S [2]. The allele frequency was 0.639 in youth from this Tribal Council [2]. Although this polymorphism is a significant risk factor for T2DM in this population, it cannot fully explain the high rates of T2DM in the Oji-Cree population of Manitoba, as 59% of youth with T2DM at DER-CA and 22% of the children with T2DM from the highest risk communities had the wild-type GG phenotype.

### 5.2. Obesity

Obesity is the most significant risk factor for the development of T2DM in childhood [5, 18]. The main causes of obesity and T2DM in the developed and the developing world include a lack of adequate physical activity and consumption of energy-dense foods containing saturated fats and sugars. In 2004, 1.1 million Canadian children (18%) aged 2–17 years were overweight and 500 000 (8%) were obese [19]. The obesogenic environment may act as another significant risk factor for youth-onset T2DM in the Oji-Cree speaking communities. In 2000, pediatric overweight and obesity was observed in 27.7% of boys and 33.7% of girls, aged 2–19 years, in Sandy Lake, Ontario [20]. In one community from the aforementioned remote regional Tribal Council, 1998 data demonstrates an obesity rate of 48% in girls and 51% in boys aged 4–19 years [12].

### 5.3. Diet

Historically, the Oji-Cree communities resided in nomadic groups surviving on the land as hunters and gatherers. This method of subsistence was physically demanding and provided food primarily from meat and fish, complemented with seasonal roots and berries. The diet was high in protein, moderate in fat, and low in carbohydrates and fibre. Permanent settlements became more common among the Oji-Cree people in the 1940s resulting in the decline of hunting and gathering as a means of survival and a dependence on prepackaged foods [21]. This new lifestyle resulted in sedentary behaviour and high-energy intakes, in particular, intakes of saturated fats and refined carbohydrates [22]. Increased consumption of foods high in simple sugars, high in saturated fats, and low in fibre has been associated with increased risk for diabetes [23, 24].

The average diet in Sandy Lake was shown by 24-hour recall to be high in saturated fats (13% of energy intake), high in cholesterol (350 mg/dL), high in refined carbohydrates (22% of energy intake), and low in dietary fibre (11 g/d) [23]. In this population, it appears that dietary behaviours are becoming progressively less healthy with subsequent generations. Adolescents consumed more refined carbohydrates and less protein, mostly in the form of junk food (potato chips, fried potatoes, hamburgers, pizza, sugar-sweetened beverages, etc.), than older generations, with older generations reporting a greater consumption of more traditional foods [23]. Finally, the increasing consumption of sugar-sweetened beverages is particularly concerning the high added sugar content, low satiety, and incomplete compensation for total energy, which increases T2DM risk independent of obesity due to the high levels of rapidly absorbable carbohydrates [25–27].

### 5.4. Physical Activity

In part, the high rates of obesity in children can also be attributed to a decrease in physical activity and fitness. Obesity and T2DM in the youth of Sandy Lake, Ontario, were associated with watching greater than 5 hours of television per day and with lesser levels of fitness [20]. Moreover, in youth it is known that greater cardiovascular fitness and lesser physical activity levels are risk factors for T2DM, independent of adiposity [28].

### 5.5. Social Determinants of Health

Additional factors influencing these lifestyles and risk of chronic disease include household factors such as poverty, unemployment, overcrowding, and low parental education levels; personal factors such as stress levels, low self-esteem, low resilience, and mental health disorders; geographic factors such as isolation, limited access to healthy food, and limited access to formal physical activity opportunities; cultural factors such as lack of inspiring community role models to promote healthy lifestyle choices, language, and traditional beliefs regarding T2DM. These same risk factors predisposing Aboriginal youth to poor diet and exercise levels also inhibit them from adequate self-management.

## 6. The Diabetic Intrauterine Environment as a New Risk Factor for Early-Onset T2DM

Although it is evident that there are many risk factors predisposing the Aboriginal youth to the development of early-onset T2DM, the rapid rise in T2DM in children remains poorly understood. Considering that permanent settlements only occurred in northern populations in Canada in the mid-twentieth century and the G319S polymorphism has been in the genome of some families for generations, it is hard to explain the 20-fold increase in incidence of pediatric T2DM in Manitoba since 1985. There is evidence to suggest a role for the intrauterine environment and epigenetic alterations related to insulin secretion and/or beta-cell mass that may influence the development of diabetes in Oji-Cree youth.

Preexisting T2DM in pregnancy has lifetime effects on the offspring including higher rates of obesity, higher rates of glucose intolerance, higher rates of T2DM, and earlier age of T2DM onset [29–32]. It is possible that this recent increase in childhood T2DM is occurring because women are developing T2DM progressively earlier, resulting in the most recent generations being the first exposed to an intrauterine environment of hyperglycemia and insulin resistance at the early stages of pregnancy. Several studies have indicated disproportionate maternal transmission of T2DM to their offspring in many diverse populations [33–38]. More specifically, adult offspring of females with T2DM when compared to offspring of males with T2DM presented with greater body weight, higher insulin levels, greater pancreatic dysfunction, and more detrimental lipid profiles, all classic signs of T2DM [39]. In the Pima Indian community of Arizona, the youth have experienced a rapid increase in prevalence of T2DM over the past 30 years and the two major factors dictating this trend appear to be an increase in weight and an increasing frequency of exposure to T2DM and gestational diabetes (GDM) *in utero *[40]. A landmark study in this population demonstrated that offspring of mothers with diabetes were much more likely to develop T2DM (45%) than those born to nondiabetic mothers (1.4%) and prediabetic mothers with GDM (8.6%) [31]. Furthermore, siblings born after the mothers' diagnosis of T2DM in this Pima community had a 3.7-fold higher risk of developing T2DM themselves than siblings born before the diagnosis of T2DM in this same mother [41]. This suggests that the causative mechanism for the increased risk of T2DM in these children is exposure to the T2DM *in utero *rather than inheritance of maternally imprinted genes harbouring a genotype which increases susceptibility for pediatric T2DM. Even in the absence of T2DM in the mother, there was a linear relationship between the weight and glucose intolerance of the offspring and their mother's glucose concentration during the third trimester [42].

In the Manitoba Oji-Cree population, preexisting T2DM in the mother was the strongest predictor of T2DM in the child (OR = 14.4), followed by GDM (OR = 4.4) [43]. In the First Nations people of Saskatchewan, a neighbouring province to Manitoba, 19% to 30% of cases of T2DM may be due to maternal GDM [44]. In the mothers with preexisting T2DM in the Manitoba Oji-Cree, 25% of their children eligible for blood glucose screening (>7 years old) had T2DM and 43% of those >10 years old had T2DM [3]. Every child with T2DM in this birth cohort had at least 1 copy of the HNF-1*α* G319S polymorphism [3]. Interestingly, in individuals with monogenic diabetes due to known, severe mutations in the HNF-1*α* gene, a significantly reduced age at diagnosis is also associated with exposure to diabetes *in utero*. This suggests that an HNF-1*α* polymorphism or mutation does not fully explain the prevalence of T2DM in this Oji-Cree cohort implicating that an epigenetic, prenatal phenomenon causing further insulin deficiency may occur [45].

## 7. Developmental Origins of T2DM

Many theories for the Developmental Origins of Health and Disease (DOHaD) have been proposed to explain prenatal programming of T2DM. In 1962, James Neel proposed the “thrifty genotype” theory. He suggested that a diabetic genotype provided an evolutionary advantage, suggesting that people with T2DM are “exceptionally efficient in the intake and/or utilization of food” and that, with changing dietary, activity, and reproductive patterns, the beneficial “thrifty” gene became a disadvantage [46]. This theory was later replaced by the “thrifty phenotype” theory of the development of T2DM. In this theory, Hales and Barker proposed that maternal malnutrition in pregnancy causes fetal malnutrition which “set in train mechanisms of fetal nutritional thrift” including underdevelopment of pancreatic beta cells, increased peripheral insulin resistance, and subsequent hyperglycemia, as well as increased adipose tissue deposition. Theoretically, in times of famine, the increased tendency to free glucose in the blood would protect the infant brain from glucose deprivation and in times of feast the infant would be efficient at delivering energy to adipose stores. Most of the supporting evidence is from studies of maternal protein malnutrition, but other nutrient deficiencies have also resulted in these same outcomes [47]. This hypothesis clearly states that the development of pathology following malnutrition in early life is dependent on the presence of later risk factors such as overnutrition, physical inactivity, and ageing. Glucose intolerance would be avoided in children and adults who continued to be malnourished because they preprogrammed their metabolism to deal with lifelong malnutrition. However, those who have a positive caloric balance would develop obesity and glucose intolerance [47].

 The thrifty phenotype theory has evolved into a more mechanistic theory developed by Gluckman and colleagues called “developmental plasticity,” the ability of an organism to develop in multiple ways depending on a particular environment [4]. Developmental plasticity requires permanent changes to expression of the fetal or infant genome in order to imprint certain characteristics for the remainder of the lifespan of the organism. Therefore, the long-term risk of disease is induced due to initial adaptive responses of the fetus to signals from the mother about her physical state [4]. This theory can be extrapolated to many diseases that show evidence of origins in early life such as T2DM, hypertension, osteoporosis, cancer, and thermoregulatory disturbances. Like the thrifty phenotype theory, development plasticity also explains the link between increased risk for T2DM and low birth weight because a poor intrauterine environment induces reduced development of skeletal muscle, increased fat deposition and reduced insulin insensitivity as a survival mechanism in a poor postnatal environment. However, the thrifty phenotype hypothesis does not attempt to explain why maternal hyperglycemia and subsequent macrosomia in the infant are a risk factor for later T2DM in the child, as excess of energy *in utero* should not induce a “thrifty” phenotype. The developmental plasticity theory, however, is able to reason that the hyperinsulinemia in the infant accompanying hyperglycemia in the mother programs the child to preferentially deposit adipose tissue leading to obesity and insulin insensitivity in later life. Thus, developmental plasticity explains why the risk for T2DM related to birth weight is observed to be U-shaped in some populations, such as the Pima Indians, with increased risk of T2DM in infants with low and high birth weights [4, 48].

## 8. Mechanisms of Development Programming

DOHaD proposes a molecular mechanism for the imprinting and long-term transmission of information. Epigenetics explains how a stable genome can be influenced to be expressed in an infinite number of ways. By DNA methylation, histone deacetylation, or through posttranscriptional modification of the genome and proteome, genes can be upregulated or downregulated without changes to the genetic sequence but with changes to the resulting phenotype. These changes can reset the fetal homeostatic set points by manifesting changes in metabolism, hormone production, hormone sensitivity, or organ development. These epigenetic modifications remain with the genome through the child's life and have been shown to be partially passed on to the next generation [49]. Whether the fetus experiences nutritional deficiency, nutritional excess, or any other stimuli *in utero*, the organism can tailor its development and genetic expression to best meet the expected future environment and these responses may not become evident until later in life [4].

Alteration of B-cell mass through prenatal programming of the fetus, by epigenetic influences or by other influences, has been proposed as a possible mechanism for the susceptibility of these offspring to T2DM. It has been observed that poor protein nutrition during pregnancy causes a decrease in beta-cell numbers in the offspring and a decrease in beta-cell function [50]. In rats, this pathology occurred due to decreased beta-cell proliferation due to a prolonged cell cycle and an increased apoptotic rate in the neonatal period [51–53]. Although research into the epigenetic mechanisms behind these beta-cell alterations is lacking, it has been demonstrated that hypomethylation of a glucocorticoid receptor gene in rat pups born after low-protein gestation led to overexpression of this receptor, increased sensitivity to glucocorticoids, and possible reduction in beta-cell mass [54]. In pregnancies complicated by T2DM, pancreas development is profoundly affected [55]. Macrosomic human neonates have highly vascularised islets of Langerhans and beta-cell hyperplasia, while IUGR neonates have reduced beta-cell mass [56]. In IUGR rat pups, the promoter of PDX-1, a gene necessary for normal beta-cell development and differentiation, undergoes deacetylation of its histone complexes with subsequent lowered expression, thus demonstrating another possible epigenetic mechanism for altered beta-cell mass following an intrauterine metabolic challenge [57]. Lastly, in pregnancies of rats complicated by T2DM, the total amino acid concentration in the fetal plasma is low; thus, offspring from pregnancies complicated by T2DM may also be susceptible to the same pancreatic alterations seen in LP offspring [58].

## 9. Conclusion and Future Directions

It is apparent that the classic risk factors for T2DM, including calorie overconsumption, macronutrient malnutrition, physical inactivity, ageing, stress, and obesity, as well as the recently discovered HNF-1*α* G319S polymorphism, coexist in the Oji-Cree population of Northern Manitoba and Northwestern Ontario. These factors may, at least partially, explain the high prevalence of pediatric T2DM in this population. However, the remarkably high prevalence of T2DM in children born to mothers with prepregnancy T2DM demands new explanations for T2DM in youth. Theories, such as the developmental plasticity, have come to embrace the intrauterine environment and early-life experience as potent influencers on the lifelong development of humans.

 Future research must further investigate the role of the maternal, prenatal environment on the development of T2DM in multiple populations. Among the Oji-Cree people, longitudinal investigation of the differences in the development of T2DM in children, depending on gender of the afflicted parent, may provide clues to this question. Possible mechanisms for the child's susceptibility to T2DM are also necessary, whether it is decreased beta-cell mass, another insulin secretion deficiency, or an insulin-resistant state, just as it is necessary to identify intrauterine factors responsible for early programming of this susceptibility, such as maternal hyperglycemia or insulin resistance. Lastly, postnatal events impacting the development of T2DM in youth must be identified, as exposure to cigarette smoking and exposure to breast feeding have been identified. It is most crucial that all these observational studies inform possible interventions during pregnancy and early life to prevent impact of T2DM in the lives of Oji-Cree people.
